# Long-Lasting Fever and Lymphadenitis: Think about *F. tularensis*

**DOI:** 10.1155/2015/191406

**Published:** 2015-11-03

**Authors:** Maria Vittoria Longo, Katia Jaton, Paola Pilo, David Chabanel, Véronique Erard

**Affiliations:** ^1^Department of Internal Medecine, Hôpital du Jura, 2800 Délemont, Switzerland; ^2^Department of Internal Medecine, Hôpital de la Broye, 1530 Payerne, Switzerland; ^3^Institute of Microbiology, University of Lausanne and University Hospital Center, 1011 Lausanne, Switzerland; ^4^Institute of Veterinary Bacteriology, Vetsuisse Faculty, University of Bern, 3012 Bern, Switzerland; ^5^Unit of Infectious Diseases, Department of Internal Medicine, HFR-Hôpital cantonal, 1708 Fribourg, Switzerland

## Abstract

We report the case of glandular tularemia that developed in a man supposedly infected by a tick bite in Western Switzerland. *Francisella tularensis* (*F. tularensis*) was identified. In Europe tularemia most commonly manifests itself as ulcero-glandular or glandular disease; the diagnosis of tularemia may be delayed in glandular form where skin or mucous lesion is absent, particularly in areas which are assumed to have a low incidence of the disease.

## 1. Clinical History

A 75-year-old man, living in a rural area of Western Switzerland, was admitted to the regional hospital in mid-July 2013 presenting with fever and myalgia lasting for 5 days. Family members reported periods of confusion over the previous 24 hours. He never traveled abroad. He is retired from the postal service and spent his free time walking in the forest. He had no direct contact with domestic or wild animals. Medical history revealed diabetes type II and hypertension.

On admission the patient was febrile with moderate agitation and confusion. There was no neck stiffness and the neurological exam was unremarkable. Except for a painless partially encrusted lesion on the left leg, the clinical exam was assessed as normal. A brain computer tomography (CT) and magnetic resonance imaging (MRI) excluded pathological finding. Cerebral spinal fluid (CSF) analysis displayed 5.2*E* + 06/L mononuclear cells (reference value < 3.0*E* + 06/L) and normal levels of protein and glucose. Blood test showed a mild elevation of CRP at 23 mg/L (reference value < 5 mg/L).

The screening for the most common infectious causes of encephalitis was performed including serological testing of* human immunodeficiency virus* (HIV), immunoblot for Lyme disease in serum and CSF, and molecular tests for* Herpes simplex virus* (HSV),* Varicella-zoster virus* (VZV),* Listeria monocytogenes,* and* Bartonella henselae* (*B. henselae*) in CSF. All were negative. CSF and blood cultures were negative.

**Figure 1:**
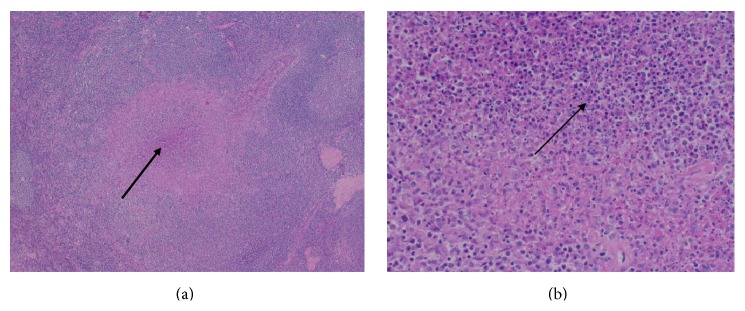
(a) HE, 4x: lymphadenitis with follicular hyperplasia and necrosis (arrow) surrounded by a histiocytic reaction. (b) HE, 20x: necrotizing and granulomatous lymphadenitis, with numerous neutrophils cells (arrow).

Patient was treated with ceftriaxone and acyclovir for 48 hours. He spontaneously recovered and was discharged home after 5 days but was readmitted a few days later with relapsing fever, confusion, and extreme fatigue. Clinical exam was unchanged. Radiological workup including chest and abdominal CT performed for investigation of fever of unknown origin (FUO) revealed enlarged left femoral lymph nodes. Because of the presumption of lymphoma, lymph node biopsy was ordered. Histological examination revealed a lymphadenitis with follicular hyperplasia including immature B lymphocytes on immunohistochemistry (IHC) and sites of necrosis containing numerous granulocytes surrounded by epithelioid cells (Figures 1(a) and 1(b)). There was no sign for oncologic or infectious process. Bacteria, including* B. henselae* and mycobacteria, were not detected by Gram, Warthin-Starry, and Ziehl-Neelsen stains. Serology for* Epstein-Barr virus* (EBV) showed past infection. Serology for* B. henselae* (IgG titer 1000, *N* < 120; IgM titer < 100, *N* < 100) was compatible with a current or an ancient infection. However* B. henselae* specific PCR performed on paraffin-embedded lymph node, as described below, was negative.

Serology for* F. tularensis*, performed by enzyme-linked immunosorbent assay (ELISA) one month after the onset of clinical manifestation, was compatible with a recent infection (IgG 300 U/mL, *N* < 10; IgM 122.4 U/mL, *N* < 10). Definite diagnostic of tularemia was confirmed by detection of* F. tularensis* DNA (600 copies/mL) extracted from formalin-fixed and paraffin-embedded tissue sections of the femoral lymph nodes, as described below. Briefly, several tissue sections were obtained from the pathologists and collected in sterile tubes. After removal of paraffin using Xylol (65°C) and further rehydration via decreasing concentrations of ethanol washes, DNA was extracted using MagNA Pure LC automated system (Roche) with the MagNA Pure LC DNA isolation kit I (Roche) and eluted in a final volume of 100 *μ*L. Francisella specific PCR, targeting the* fopA* gene as previously reported [1] and* B. henselae* specific PCR targeting the* htr A* gene were performed.

The patient recovered after completion of 3 weeks of ciprofloxacin treatment.

## 2. Discussion

Tularemia is a zoonosis mainly occurring in the Northern Hemisphere. Humans may acquire the disease through the handling of infected animals, ingestion of contaminated food or water, inhalation of infective aerosols, and hematophagous arthropod bites [2].* F. tularensis,* the agent of tularemia, comprise 3 subspecies:* F. tularensis* subspecies (subsp.)* holarctica, tularensis,* and* mediasiatica. F. tularensis* subsp.* tularensis* and* F. tularensis* subsp.* holarctica* are clinically relevant for humans.* F tularensis* subsp.* tularensis* is only present in North America, while the subspecies* holarctica* is widespread in the whole Northern Hemisphere [3]. Infection with* F. tularensis* leads to 6 major clinical manifestations primarily reflecting the route of infection and comprising ulceroglandular, glandular, oculoglandular, oropharyngeal, pneumonic, and typhoïdal syndrome. However, tularemia may present with nonspecific symptoms and routine laboratory testing, hampering its rapid diagnosis, especially in new endemic region. Ordinarily the onset of disease is abrupt occurring in an average of 3 days but ranging from 3 to 30 days after exposure. Fever, chills, and headache malaise are frequent. Persistent high fever is common. Ulceroglandular form is the most common form in Europe, presenting commonly as a localized lymphadenopathy. The initial skin lesion appears as a cutaneous ulcer, usually solitary and evolving over the course of the disease in “encrusted,” “ulcerous,” or “pustular” wound [4].

The histologic examination of the regional lymph node revealed a necrotizing and granulomatous lymphadenitis [5]. Granulomatous lymphadenitis may be representative of infectious or noninfectious processes. Noninfectious causes encompass sarcoidosis or sarcoid-like reaction observed in many underlying diseases. Infectious lymphadenitis is histologically categorized into suppurative or nonsuppurative, according to the presence or absence of granulocytes in necrotic area. Follicular hyperplasia, B lymphocytosis, histiocytic reaction, and granuloma with numerous granulocytes in central necrosis are characteristically depicted in adenitis associated with* F. tularensis* and* B. henselae* infection [6]. In opposite, nonsuppurative adenitis, characterized by granulocyte-free necrosis, is described in* Mycobacterium tuberculosis* and* Toxoplasma gondii* infections. Thus, in absence of available tissue for culture, histological description may presume the involved microorganism and suggest additional tests to establish a definite diagnosis. Tularemia is generally diagnosed either by serological tests comprising microagglutination and enzyme-linked immunosorbent assays (ELISA), by isolation of* F. tularensis,* or by performing a specific* F. tularensis* PCR from clinical material including wound drainage, lymph node aspirate, sputum, and blood. The isolation of the agent of tularemia by culture or the detection of* F. tularensis* DNA by PCR on fresh and frozen tissues is particularly useful in the early phase of the disease when antibodies are not yet present and the treatment is more effective [7].

In the present case the diagnosis was evoked lately in spite of suggestive clinical and histological clues including febrile lymphadenitis, encrusted cutaneous lesion attributed to arthropod bite, and suppurative granulomatous adenitis. While arthropod-borne diseases such as tick-borne encephalitis and Lyme disease are well known by physicians, tularemia is still rarely evoked by doctors in Switzerland, despite an increasing number of reported cases since 2008 [8–11]. A study published in 2000 reported that out of 6071* Ixodes ricinus* ticks collected on Swiss Army training grounds in five regions of Switzerland, 0.12% harbored* F. tularensis* DNA [12].

Nervous system abnormalities are uncommon manifestations of tularemia and have been exceptionally reported as meningitis and encephalitis, probably following meninges seeding during untreated bacteremia [13–16]. Meningitis or encephalitis may occur following all of the 6 syndromes caused by* F. tularensis*, developing in a median of 5 days, ranging from 3 to 30 days after the onset of initial manifestation [17]. CSF analysis usually reveals mononuclear pleocytosis, variable level of protein and glucose, and generally negative Gram stain [17, 18]. Based on unremarkable CSF analysis and CNS radiologic evaluation, the cause of the confusion and the contribution of* F. tularensis* in the abnormal behavior observed in our patient could not be established.

Aminoglycosides (streptomycin and gentamicin), fluoroquinolone, and tetracyclines are the drugs commonly used to treat tularemia. Until recently macrolides were considered effective in cases acquired in Switzerland and Western European countries. Azithromycin was even considered as first line treatment option during pregnancy in France [19]. With the growing interest in* F. tularensis* biology and the advent of novel molecular technologies, the genome of an increasing number of strains is sequenced leading to the discovery of new subgroups [20–22]. In Europe, strains of* F. tularensis* subsp.* holarctica* belonging to groups B.13 and B.FTNF002-00 are predominant [23]. Until recently, only strains of group B.FTNF002-00 were isolated from human and animal tularemia cases in Switzerland. However, Origgi and colleagues reported that strains belonging to group B.13 are circulating in Switzerland in the same areas than strains of group B.FTNF002-00 at least since 2012 [24]. Because of the antibiotic resistance profile of this group, this discovery is relevant for clinical practice. Indeed, while the strains belonging to group B.FTNF002-00 are sensitive to erythromycin, the strains of group B.13 are not. Macrolides should not be recommended to treat cases acquired in Switzerland without prior typing of the strains. The minimal inhibitory concentration (MIC) values of antibiotic drugs relevant to clinical use (concentrations tested: gentamycin [0.12–16 mg/L], tetracycline [0.25–16 *μ*g/mL], erythromycin [0.5–32 *μ*g/mL], chloramphenicol [2–32 *μ*g/mL], and ciprofloxacin [0.06–4 *μ*g/mL]), were performed on a panel of 24 strains isolated between 1996 and 2013 from human and animal cases of tularemia in Switzerland. Ciprofloxacin was found to show the lowest MIC values and prevented growth of all strains at 0.06 *μ*g/mL [24]. These results are in accordance with previous studies [25–27]. Gentamicin used to be the reference treatment for tularemia. Because of its IV formulation and side effects, its use is currently restricted to severe tularemia cases. Fluoroquinolone and tetracyclines, especially ciprofloxacin and doxycycline, respectively, are advocated as first line drugs for patients with diseases of mild to moderate severity. Treatment failures and relapses may occur in up to 10% of patients treated with a fluoroquinolone and even more frequently when a tetracycline is administered; however the natural acquisition of antibiotic resistance has not been proven so far.

Tularemia should be considered in presence of fever and enlarged lymph node in countries where tularemia is endemic or sporadic cases in animals and humans occur. Localization of adenitis often reflects the route of infection. Primary respiratory tract infections generally involve mediastinal and/or hilar lymph nodes and skin inoculation implicate regional lymph nodes, while oropharyngeal and conjunctival contamination induce adenopathy in cervical, preauricular, and submandibular regions. Meticulous examination of skin and mucosa to identify the portal of entry and inquiry on recent tick bite may guide the diagnosis. Oropharyngeal tularemia should be excluded before making the diagnosis of tuberculosis in presence of cervical lymphadenitis, especially if histology displays suppurative necrotizing granulomatous lymphadenitis, and tularemia should be considered in patients with a history of fever and ulcerative skin lesion following arthropod bite.

In the presence of enlarged lymph node and lung infiltrate careful interrogation on exposition to contaminated dust or sick animals may orientate the diagnosis toward tularemia or Q fever.

Due to its convenient use, its rare side effects, and its lowest MIC compared to that of other effective antibiotics, quinolone may be considered as the first line treatment in nonsevere tularemia.
